# Two new species of the genus *Methocha* from Laos (Hymenoptera, Tiphiidae)

**DOI:** 10.3897/zookeys.775.24945

**Published:** 2018-07-17

**Authors:** Keisuke Narita, Toshiharu Mita

**Affiliations:** 1 Entomological Laboratory, Graduate School of Bioresourse and Bioenvironmental Sciences, Kyushu University, Motooka 744, Fukuoka, Japan; 2 Entomological Laboratory, Faculty of Agriculture, Kyushu University, Motooka 744, Fukuoka, Japan

**Keywords:** Cicindelinae, new record, parasitoid, taxonomy

## Abstract

Two new species of *Methocha*, *M. cariniventris* and *M. granulosa* are described from Laos.

## Introduction

The genus *Methocha* Latreille contains 86 species throughout the world except for the Australian region (Krombein 1982, Tsuneki 1986, Agnoli 2011, Kimsey 2011, Terayama and Mita 2015). This genus shows considerable sexual dimorphism, with winged males and wingless, ant-like females. Although the life history of most species is still unknown, they are considered to be parasitoids of Cicindelinae beetle larva (Coleoptera, Carabidae) (Adlerz 1903, 1906, Bouwman 1909, Champion and Champion 1914, Williams 1919, Pagden 1926, Main 1927, Yasumatsu 1931, Iwata 1936, Krombein 1982).

The tiphiid subfamily Methochinae includes two genera, *Methocha* and *Karlissa* Krombein (Kimsey 1991, Brothers and Carpenter 1993). Some molecular phylogenetic studies suggested that Methochinae and Tiphiinae were paraphyletic, and Methochinae were moved to Thynnidae (Pilgrim et al. 2008, Debevec et al. 2012). However, their close relationship was rather strongly supported by recent studies based on transcriptomes (Peters et al. 2017) and UCEs (Branstetter et al. 2017). Although family concept remains disputable, here we follow the classification proposed by Aguir et al. (2013).

*Methocha* are most diverse in the Oriental region. Up to now, 52 species have been recorded (Krombein 1982, Tsuneki 1986, Agnoli 2011, Terayama and Mita 2015), and 15 species are known from Southeast Asia, including Malaysia, Philippines, and Indonesia. However, none of them have been recorded from Laos. We had an opportunity to examine some specimens of *Methocha* from Laos collected by biotic surveys on 2009 and 2011, and found several interesting specimens. These specimens were classified into two new species by the following characters; shape of the clypeus, sculpturing of the pronotum, the mesopleuron, and the propodeum, and infuscation of the wings.

## Materials and methods

Specimens examined are deposited in the Entomological Laboratory, Faculty of Agriculture, Kyushu University, Fukuoka, Japan (ELKU). The terminology follows those of Richards (1977) and Krombein (1982). The following abbreviations are used in descriptions:

**BL** body length;

**HL** head length;

**HW** head width;

**WF** width of frons;

**POL** length between posterior ocelli;

**AOL** length between anterior ocellus and posterior ocellus;

**DAO** diameter of anterior ocellus;

**EL** eye length;

**FWL** fore wing length;

**ML** mesosoma length;

**MW** mesosoma width;

**T** metasomal tergite;

**S** metasomal sternite;

**AMW** anterior width of T1;

**MTL** T1 length;

**PMW** posterior width of T1.

Observations were made on an Olympus SZX-1212 stereomicroscope. Photo images were taken by a Canon EOS-60D with a Canon MP-E 65 mm 1–5× a Macro lens and processed by image stacking software, Combine ZM (Halrey 2010).

## Results and discussion

### Methocha cariniventris sp. n.

Taxon classificationAnimaliaHymenopteraThynnidae

http://zoobank.org/EDCC6019-5CB6-4F50-B81D-3E645062DD7A

Figs 1–3 , 4 , 5–12

#### Material examined.

Holotype, ♂, LAOS: Mt. Phou Pan Gnai, Houa Phan Prov., VI. 2009, H. Kojima leg. (ELKU).

#### Diagnosis.

This species is characterized by combination of the following characters: the distally wide mandibles; the translucent distal margin of the clypeus; the areolate propodeum; infuscate near apical 1/2 of the wings. This species is similar to *M. foveiventris* Lin, 1966 and *M. punctata* Williams, 1919 in the body size, but it can be distinguished by the striate propodeum (reticulate rugose in *M. foveiventris*) and the translucent distal margin of the clypeus (opaque in *M. punctata*).

**Figures 1–3.**
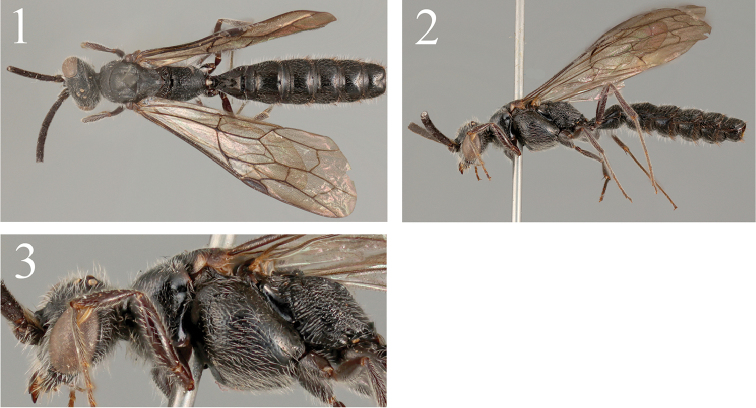
*Methocha cariniventris* sp. n., male, holotype. **1** Dorsal habitus **2** lateral habitus **3** mesosoma, lateral view.

#### Description.

**Figure 4.**
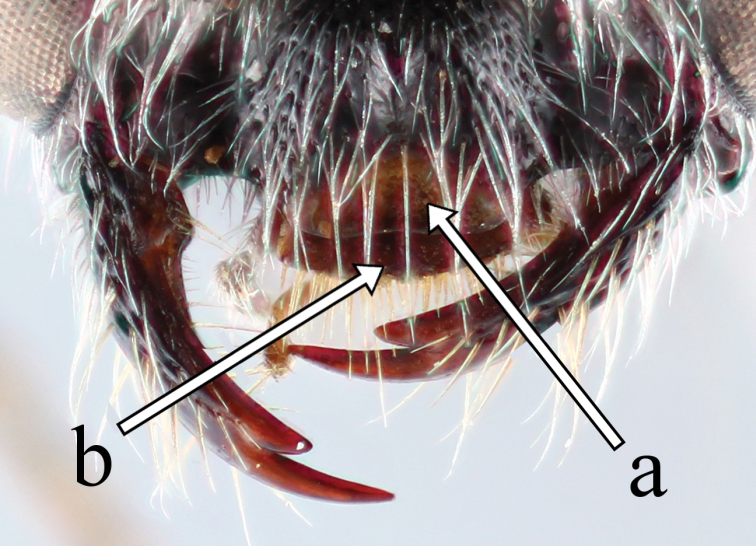
Frontal head of *Methocha cariniventris* sp. n. male, holotype. Arrow a indicating translucent distal apex of clypeus; arrow b indicating labrum.

**Figures 5–12.**
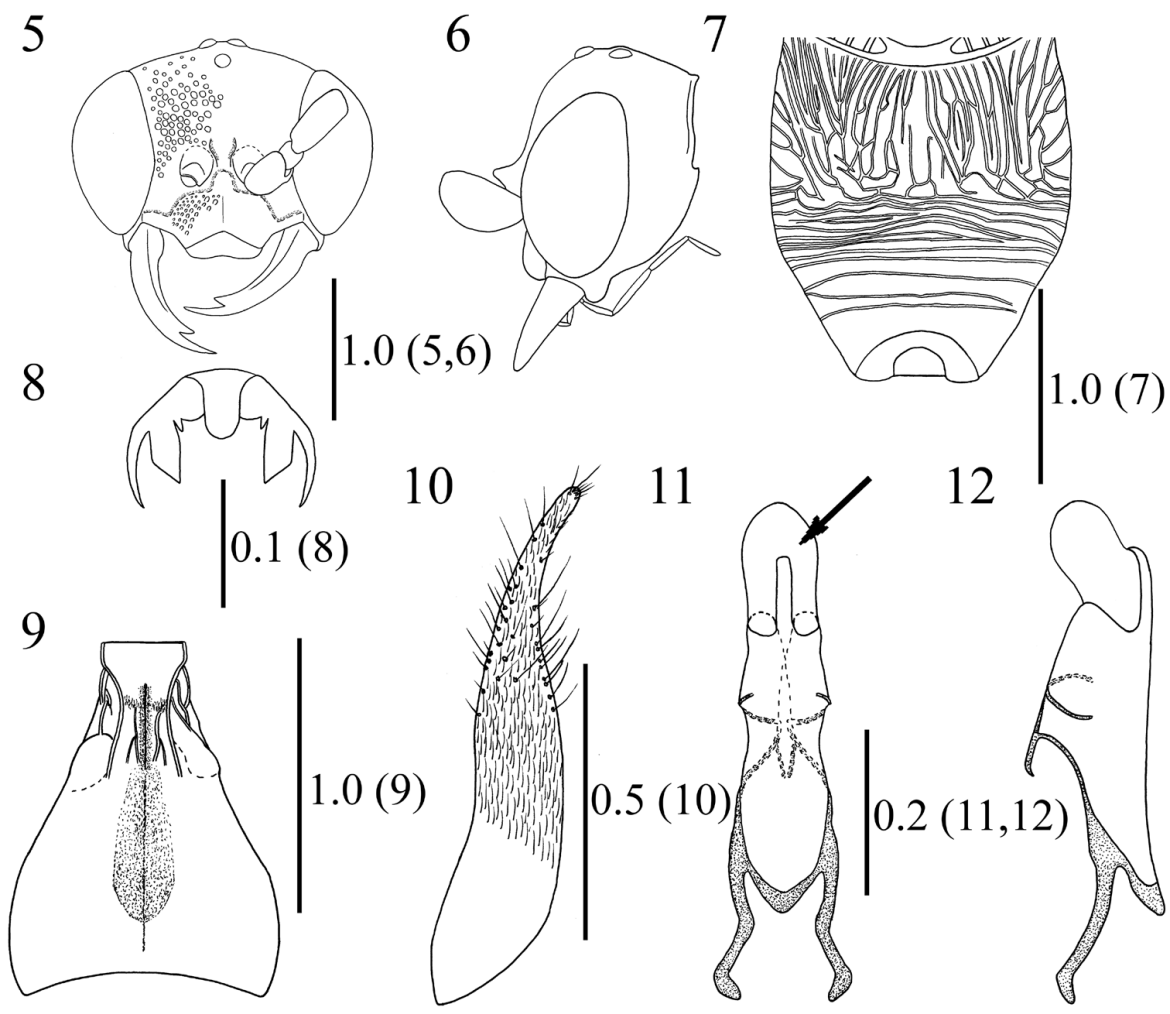
*Methocha cariniventris* sp. n., male, holotype. **5** Head, frontal view **6** head, lateral view **7** propodeum **8** hind claw **9** T1, dorsal view **10** right paramere, lateral view (right side is dorsal) **11** aedeagus, dorsal view, arrow indicating apical membranous lobe **12** aedeagus, lateral view (right side is dorsal). Scale bars are given in mm.

Male. *Head* (Figs 5, 6). Height 0.7 times as long as wide in frontal view; temple weakly rounded; ocelli forming regular triangle, POL: AOL: DAO = 1.0: 1.0: 0.6; vertex punctate: punctures smaller and sparser than those on frons, 1.0–5.0 puncture diameters apart, with interspaces smooth; frons densely punctate: punctures 0–1.0 puncture diameters apart, deeply excavated above antennal lobes; antennal lobes developed; gena punctate as vertex; clypeus (Figure 4) densely covered with small punctures, distinctly convex, semicircular laterally, apical 1/5 translucent, subtruncate; mandible not narrowed distally, lower tooth longer than upper tooth; maxillary palpus shorter than length of pronotum, length (width) of segments II–VI showing following ratio: 1.0 (0.3): 2.0 (0.3): 3.0 (0.3): 2.4 (0.3): 2.4(0.3); flagellum weakly flattened, length (width) of flagellomeres I–III showing following ratio: 1.7 (1.0): 2.3 (1.0): 2.4 (1.0).

*Mesosoma* (Figure 3). Pronotal disc punctate: punctures 1.0–3.0 puncture diameters apart, with interspaces smooth, anterior transverse carina absent, lateral surface punctate as dorsal surface, transversely faintly rugulose medially; mesoscutum punctate: punctures smaller and denser anteriorly, with interspaces smooth, sometimes coriaceous, larger and sparser medially; notauli indicated by transversely striate wide depression, reaching posterior 1/3, posterior margin effaced; mesoscutellum elevated, dorsal surface sparsely, lateral slope finely punctate, with interspaces smooth, lateral lower surface transversely carinate; mesepisternum convex, coarsely punctate: punctures 1.0 puncture diameter apart, with interspaces smooth, narrow surface above episternal sulcus densely punctate: punctures 0–0.5 puncture diameters apart, episternal sulcus deep, precoxal sulcus obscure, faintly depressed; metanotum elevated, smooth, laterally striate, anterior margin with deep crescentic depression; metapleural region antero-dorsally smooth, posteriorly rugose; dorsal surface of propodeum (Figure 7) irregularly longitudinally areolate, with interspaces smooth, lateral surface coarsely puncto-reticulate, posterior surface transversely rugose.

*Legs*. Hind coxa dorsally carinate, parabolically elevated in lateral view; claws (Figure 8) curved at apical 1/2, tridentate, subapical tooth stout, truncate, basal tooth minute, less than 1/5 as long as subapical tooth.

*Metasoma*. Tergites smooth with sparsely located punctures and setae; AMW: MTL: PMW = 1.0: 2.9: 2.5; T1 (Figure 9) with pair of strong carinae present before spiracle, rugose between carinae, medial furrow present around anterior 4/5, shallower and wider posteriorly; anterior transverse depression of T2 costate, T3–T7 without carinae, smooth; posterior margin of S2–S6 with row of brown stout setae; punctures on S7 smaller than those on S1–S6, 1.0–2.0 punctures diameters apart, posterior margin of S7 not cleft; ventral surface of hypopygium longitudinally striate, hairy; paramere (Figure 10) narrow, apical 1/4 slender; aedeagus (Figs 11, 12) with apical membranous lobe, apical hook faintly curved ventrad, aedeagal apodeme moderately curved ventrad except distal apex.

*Color*. Black; scape brown; distal margin of clypeus translucent yellow; maxillary palpus dark yellow; apical 1/3 of mandible dark brown; tegula dark brown; wings weakly tinged with brown, with apical 1/2 strongly infuscate; legs dark brown.

*Measurements*. BL 11.0 mm, HL 1.5 mm, HW 2.1 mm, WF 1.0 mm, EL 1.1 mm, ML 3.9 mm, MW 1.7 mm, FWL 4.7 mm.

#### Etymology.

The name is derived from the propodeal sculpture.

#### Distribution.

Laos: Houa Phan Province.

#### Remarks.

The female is unknown.

### Methocha granulosa sp. n.

Taxon classificationAnimaliaHymenopteraThynnidae

http://zoobank.org/D38A72B4-9143-4AD6-94CD-A7876FC283FE

Figs 13–15 , 16–23

#### Materials examined.

Holotype, ♂, LAOS: 19°33’N, 103°41’E, 570m alt., Hot spring near Ban Ban, Houa Phan Prov., 4. IV. 2011, T. Mita leg. (ELKU); Paratypes, 3♂, with same data as holotype (ELKU).

#### Diagnosis.

This species is characterized by combination of the following characters: the distally wide mandibles; the translucent distal margin of the clypeus; the distinct pleuro-propodeal suture; the striate and granulate propodeum; the faintly infuscate wings. This species can be easily distinguished from other Asian species by the above-mentioned characters.

#### Description.

Male. *Head* (Figs 16, 17). Height 0.7–0.9 times as long as wide in frontal view; temple weakly rounded; ocelli forming regular triangle, POL: AOL: DAO = 1.0: 1.0: 0.6–0.8; vertex punctate: punctures smaller and sparser than those on frons, 1.0–4.0 puncture diameters apart, with interspaces smooth; frons densely punctate: punctures 0.5–1.0 puncture diameters apart, deeply excavated above antennal lobes; antennal lobes developed; gena punctate as vertex; clypeus covered with small punctures, distinctly convex, apical 1/5 translucent, with apex subtruncate; mandible not narrowed distally, lower tooth longer than upper tooth; maxillary palpus slightly shorter than length of pronotum, length (width) of segments I–VI showing following ratio: 4.2 (1.2): 3.3–5.0 (1.2): 5.0–6.7 (1.2): 6.7–8.3 (1.2): 5.8–6.7 (1.2): 5.0–8.3 (1.2); length (width) of flagellomeres I–III showing following ratio: 2.0–2.4 (1.6): 2.6–2.9 (1.6): 2.7–3.0 (1.6).

*Mesosoma* (Figure 15). Pronotal disc more or less covered with shallow punctures, with interspaces smooth, anterior transverse carina absent, lateral surface smooth; mesoscutum punctate: punctures denser and smaller around anterior 1/3, larger and sparser at posterior 2/3, punctures sometimes entirely sparser; notauli indicated by transversely striate wide depression, reaching posterior 1/2 to 2/3, posterior margin effaced; mesoscutellum elevated, dorsal surface sparsely, lateral slope finely punctate, sometimes almost entirely impunctate, lateral lower surface transversely rugose by weak carinae, sometimes almost smooth; mesepsternum convex, smooth, with several small punctures, episternal sulcus deep, precoxal sulcus obscure, faintly depressed; metanotum elevated, smooth, laterally striate, anterior margin with deep crescentic depression; metapleural region smooth, postero-dorally longitudinally rugose, with pleuro-propodeal suture distinct, arising from lower pit to propodeal spiracle; dorsal surface of propodeum (Figure 18) more or less longitudinally rugose on anterior 1/3, with interspaces granulate, medial 1/3 transversally rugose, with interspaces granulate, lateral surface sparsely punctate: punctures 2.0–4.0 punctures diameters apart, with interspaces smooth, around pleural-propodeal suture rugose.

**Figures 13–15.**
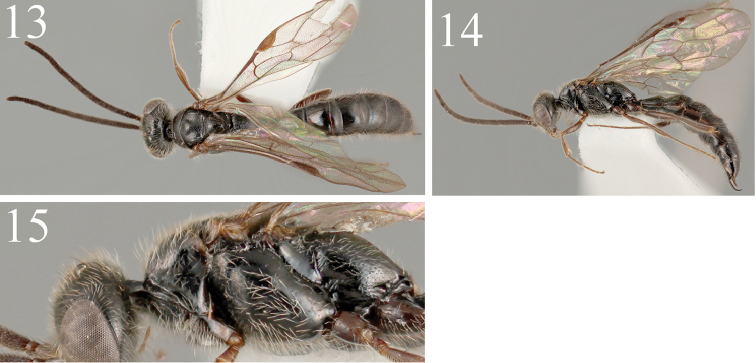
*Methocha granulosa* sp. n., male, paratype. **13** Dorsal habitus **14** lateral habitus **15** mesosoma, lateral view.

*Legs*. Hind coxa dorsally carinate, parabolically elevated in lateral view; claw (Figure 19) strongly curved at apical 1/3, tridentate, subapical tooth stout, truncate, basal tooth minute, less than 1/5 as long as subapical tooth.

*Metasoma*. Tergites smooth with sparsely located punctures and setae with fine punctures; ATL: MTL: PTL = 1.0: 2.9–3.3: 2.4–3.0; T1 (Figure 20) with pair of strong carinae present before spiracle, rugose and weakly excavated medially between carinae, medial furrow present on anterior 4/5, shallower posteriorly; anterior transverse depression of T2–T4 costate, T5–T7 without carinae, smooth; posterior margin of S2–S6 with row of brown setae; S7 with large punctures: punctures 1.0 puncture diameter apart, apical margin semicircularly cleft; ventral surface of hypopygium rugose, hairy; paramere (Figure 21) narrow, apical 1/3 slender and nearly straight; aedeagus (Figs 22, 23) with apical membranous lobe, apical hook strongly curved ventrad; aedeagal apodeme almost straight except distal apex laterally.

**Figures 16–23.**
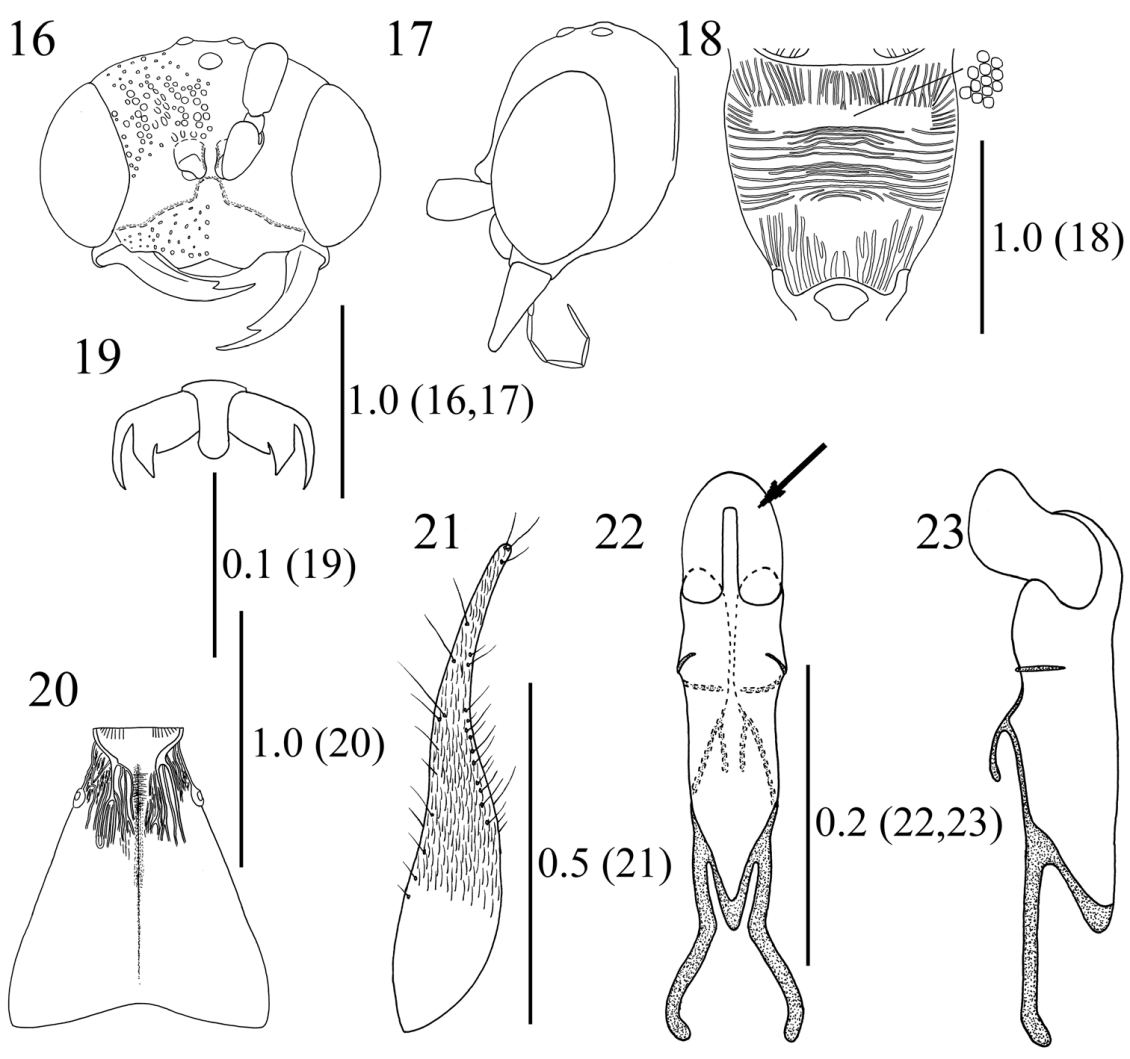
*Methocha granulosa* sp. n., male, holotype. **16** Head, frontal view **17** head, lateral view **18** propodeum **19** hind claw **20** T1, dorsal view **21** right paramere (right side is dorsal), lateral view **22** aedeagus, dorsal view, arrow indicating apical membranous lobe **23** aedeagus, lateral view (right side is dorsal). Scale bars are given in mm.

*Color*. Black; antenna dark brown; distal margin of clypeus translucent yellow; maxillary palpus dark testaceous; apical 1/4 of mandible dark brown; tegula brown; wings faintly infuscate; legs brown.

*Measurements*. BL 7.00–7.5 mm; HL 0.9–1.3 mm, HW 1.2–1.4 mm, WF 0.6–0.7 mm, EL 0.7–0.8 mm, ML 2.3–2.7 mm, MW 1.0–1.3 mm, FWL 4.5–6.0 mm.

#### Etymology.

The name is derived from the propodeal sculpture.

#### Distribution.

Laos: Houa Phan Province.

#### Remarks.

The female is unknown.

## Supplementary Material

XML Treatment for Methocha cariniventris

XML Treatment for Methocha granulosa
